# Oxidative stress and antioxidant imbalance in ovulation disorder in patients with polycystic ovary syndrome

**DOI:** 10.3389/fnut.2022.1018674

**Published:** 2022-10-28

**Authors:** Wenqian Li, Chang Liu, Qingmei Yang, Ying Zhou, Min Liu, Hongying Shan

**Affiliations:** ^1^Department of Reproductive Medicine, First Affiliated Hospital, School of Medicine, Shihezi University, Shihezi, China; ^2^Department of Obstetrics and Gynecology, Reproductive Medical Center, Peking University Third Hospital, Beijing, China

**Keywords:** polycystic ovary syndrome, oxidative stress, hyperandrogenism, insulin resistance, ovulation disorder, antioxidants, overweight/obesity

## Abstract

Polycystic ovary syndrome (PCOS) is a common reproductive endocrine disease that is characterized by oligo-ovulation or anovulation, hyperandrogenism, and polycystic ovaries observed using ultrasound with high clinical heterogeneity. At present, the etiology of PCOS is not clear but is thought to be related to genetic, metabolic, endocrine and environmental factors. Hyperandrogenism interacts with insulin resistance and overweight/obesity, forming a vicious cycle of mutual promotion and participating in the occurrence and progression of PCOS. Oxidative stress (OS) refers to the imbalance between the oxidation system and antioxidation system in the human body, which is associated with the occurrence and development of various diseases. Recent studies have shown that OS may be closely related to ovulation disorders in PCOS, and antioxidants can improve the oxidative stress state of PCOS. However, previous studies did not examine the effect of the interaction between OS and hyperandrogenism, insulin resistance or overweight/obesity on ovulation disorders in PCOS. This article reviews the interaction between OS and hyperandrogenism, insulin resistance and overweight/obesity; the effects of OS, hyperandrogenism, insulin resistance and overweight/obesity on ovulation disorders in PCOS; and the application of antioxidants in PCOS.

## Introduction

Polycystic ovary syndrome (PCOS) is a clinical syndrome characterized by anovulation or oligo-ovulation, hyperandrogenism and polycystic ovaries, affecting the female reproductive system, endocrine metabolism and psychological status. The overall prevalence rate is 6%-10%(1), and PCOS is an important cause of ovulation disorder infertility. The clinical manifestations of PCOS have high heterogeneity. Most patients also have endocrine and metabolic disorders, such as insulin resistance, obesity, dyslipidemia and compensatory hyperinsulinemia. Because its pathogenesis has not been fully elucidated, PCOS has gradually become a research hotspot in recent years. Previous studies have confirmed that genetic factors, environmental factors and epigenetics are related to the occurrence of PCOS. Hyperandrogenemia and insulin resistance, as the pathophysiological basis of PCOS, play an important role in its occurrence and development. At the same time, overweight/obesity can make PCOS symptoms worse by amplifying various features. Oxidative stress (OS) refers to the imbalance between oxidation and antioxidation in the human body. The accumulation of excessive oxidative active substances leads to tissue cell dysfunction. It has been confirmed that OS is involved in the regulation of the occurrence and development of various diseases, especially PCOS. How does OS regulate hyperandrogenism, insulin resistance and overweight/obesity, leading to ovulation disorders in PCOS patients? This paper reviews this issue.

## Polycystic ovary syndrome

Polycystic ovary syndrome (PCOS) is one of the most common reproductive endocrine diseases. The incidence of PCOS is approximately 6-10% in the population (1). PCOS has high clinical heterogeneity: it can have no obvious clinical symptoms, or the symptoms can include irregular menstruation, infertility, androgen excess, obesity and so on (2). At the same time, PCOS is often accompanied by embryonic high androgen exposure, endocrine and metabolic disorders, chronic inflammation, immune abnormalities, oxidative stress and other risk factors. The risk of cardiovascular and cerebrovascular diseases, type II diabetes, endometrial hyperplasia, endometrial cancer, metabolic syndrome and neurodegenerative diseases is increased in the long term, which seriously affects the physical and mental health of women (3–6). At present, the Rotterdam diagnostic criteria established in 2003 are internationally accepted. PCOS diagnosis requires two of the following three items: anovulation or oligo-ovulation, hyperandrogenism, and polycystic ovaries. Other causes that may lead to hyperandrogenism, such as congenital adrenal hyperplasia and Cushing syndrome, can be diagnosed as PCOS (7). To date, there is no unified and clear conclusion on the mechanism of PCOS in the medical field, but scholars generally believe that PCOS is the result of the interaction between environmental factors and genetic factors (8).

## Oxidative stress

Oxidative stress (OS) refers to the imbalance between the oxidation system and antioxidation system in the human body. The accumulation of active oxidation substances in the body causes protein and DNA damage and lipid peroxidation, which further lead to cell dysfunction (9). Reactive oxygen species (ROS) and reactive nitrogen species (RNS) are two basic types of oxidative active molecules in the body. Typical representative types of ROS include superoxide anion (O_2_^–^), hydroxyl radical (OH) and hydrogen peroxide (H_2_O_2_). The main source of ROS *in vivo* is oxidative phosphorylation of mitochondria, and the secondary sources are cytochrome P450 enzyme, peroxisome, xanthine oxidase and activated inflammatory cells (10). RNS includes nitric oxide (NO) and nitrogen dioxide (NO_2_)(11). Ionizing radiation, metal ions, and pollutants in the atmosphere are important sources of exogenous oxidizing active molecules (12). The body’s aging and inflammatory response can also promote the production of oxidative active molecules. ROS and RNS have dual effects on cells. Low and medium concentrations of oxidative active molecules can participate in a variety of physiological functions (such as the body’s anti-infection process), or they can be used as a second messenger for cells that are responsible for intracellular signal transduction, gene expression regulation, and cell proliferation, differentiation and apoptosis control (13). Antioxidants in the body include antioxidant enzymes and non-enzymatic antioxidants. Antioxidant enzymes include superoxide dismutase (SOD), catalase, and glutathione (GSH) peroxidase, and non-enzymatic antioxidants include vitamin C, vitamin E, GSH, taurine, hypotaurine, zinc, selenium, carotenoids, and metal binding proteins. All of them have the ability to scavenge oxidative active molecules and maintain the oxidant/antioxidant balance (14). Excessive oxidative active molecules can affect the function of biological molecules by modifying protein molecules, causing lipid peroxidation and DNA damage. At the same time, when the body’s antioxidant defense function is not enough to remove a large number of oxidized active molecules, the imbalance between oxidant and antioxidant levels will eventually lead to OS, resulting in cell damage and causing a variety of biological processes (15).

## Oxidative stress in polycystic ovary syndrome

Oxidative stress is associated with the occurrence and development of various diseases, such as cardiovascular and cerebrovascular diseases, neurodegenerative diseases, multiple cancers and type II diabetes, which seriously threaten human health (16–20). Previous studies have shown that OS is closely related to the occurrence and development of PCOS. Papalou et al. (21) analyzed the role of OS in PCOS and suggested that the accumulation of oxidative active molecules in PCOS patients cannot be offset by antioxidant defense function. OS is an important part of PCOS pathophysiology and regulates the occurrence and development of PCOS with other pathogenic factors. When studying the relationship between circulating apoptosis markers and oxidative stress in PCOS patients, Uyanikoglu et al. (22) found that in the blood of PCOS patients, the total oxidation state, total antioxidation state and oxidative stress index were all higher than those of healthy women in the control group, and the total oxidation state was higher than the total antioxidation state, indicating that there was an imbalance between oxidants and antioxidants in PCOS patients. Similarly, the meta-analysis results of Murri et al. (23) suggested that the levels of OS markers in the blood of PCOS patients, such as homocysteine, asymmetric dimethylarginine, malondialdehyde (MDA) and SOD, were significantly higher than those in the control group, while the levels of antioxidant markers, such as GSH and paraoxonase-1, were significantly lower than those in the control group.

## Oxidative stress, Hyperandrogenemia, insulin resistance and ovulation disorder in polycystic ovary syndrome

### Hyperandrogenism

Hyperandrogenism (HA) is one of the main clinical features of PCOS. The proportion of patients with elevated serum androgen is 60-75% (24). Elevated serum testosterone level may be related to the occurrence of PCOS (25). Gonadotropin-releasing hormone (GnRH) pulse secretion in females occurs during adrenarche. By regulating the gonadal axis, the secretion of adrenocorticotropic hormone (ACTH) and luteinizing hormone (LH) is increased (26), thereby stimulating the secretion of excessive androgens from the adrenal gland and ovary. Because of the high reactivity of 17-ketosteroid to adrenocorticotropic hormone, the synthesis of androgen in the adrenal gland is active, which can significantly increase the level of serum androgen (27). Therefore, adrenal androgen secretion appears earlier than ovarian steroid secretion, which may be the initial source of female androgen (28). The increase in LH pulse secretion frequency can promote the production of excessive androgen in theca-interstitial cells. Although progesterone can slow down the pulse frequency of GnRH by inhibiting the discharge of GnRH neurons, excessive androgen can still accelerate the pulse frequency of LH by reducing the effect of progesterone (29), causing the secretion of androgen in ovarian tissue. In addition, PCOS patients usually have insulin resistance, and their insulin levels are elevated (30). Insulin can directly or synergistically act with LH to stimulate the proliferation of theca-interstitial cells and inhibit the production and secretion of serum sex hormone binding globulin (SHBG), thereby improving the level and bioavailability of serum androgen (31, 32). At the same time, a high insulin level can inhibit the production of insulin-like growth factor-I (IGF-I) receptor, indirectly increase the level and biological activity of IGF-I, and facilitate cooperation with LH to indirectly stimulate androgen production (33).

### Insulin resistance

Insulin resistance (IR) refers to the physiological concentration of insulin required to promote the ability of peripheral tissue cells to use glucose, and 50-80% of PCOS patients have varying degrees of IR (34). The combination of insulin and its receptor causes tyrosine phosphorylation of insulin receptor substrate (IRS), activates signal transduction pathways, triggers a series of cascade reactions, and realizes its physiological function (35). Of these effects, tyrosine phosphorylation of the IRS plays an important role in the process of insulin signal transduction. Defects in insulin signal transduction are the main cause of IR in PCOS patients. In addition, PCOS tissue (especially adipose tissue) cells are infiltrated by macrophages so that the body has a low degree of inflammation (36). Macrophages in adipose tissue can produce a large number of proinflammatory factors, such as tumor necrosis factor-α (TNF-α) and interleukin (IL)-1. They play a role in the paracrine and endocrine mechanisms, activate inflammatory pathways in insulin target cells, activate serine kinases in adipocytes, and cause serine phosphorylation of IRS-1, which hinders the signal transduction of insulin (37–39). The activation of some serine kinases can induce the production of suppressor of cytokine signaling 3 (SOCS3), thereby inhibiting the expression of IRS-1 and tyrosine phosphorylation, interfering with insulin activity, reducing the sensitivity of tissue cells to insulin, and ultimately leading to IR (40, 41).

### Interaction between oxidative stress, Hyperandrogenemia, and insulin resistance: A vicious cycle

A large number of studies have confirmed that PCOS patients are in a long-term state of oxidative stress imbalance, and OS has become a key factor in the pathogenesis of PCOS (23, 42). As an important pathophysiological basis of PCOS, HA and IR can be induced or aggravated under OS imbalance. In PCOS, a glucose diet can induce an increase in OS levels and activate nuclear transcription factor-kB, causing the body to be in a state of chronic low-grade inflammation and promoting the expression of various inflammatory factors, such as TNF-α and IL-6 (43, 44). On the one hand, TNF-α in the ovary can stimulate the proliferation of theca-interstitial cells (45), and the expression of cytochrome P450-17α-hydroxylase (CYP17) is upregulated in the inflammatory state, increasing the production of androgen (46). On the other hand, the expression of hepatocyte nuclear factor-4α (HNF-4α) in the OS state is downregulated, which increases the biological activity of androgen by inhibiting the expression and secretion of SHBG and eventually leads to HA (47). HA in PCOS can improve the sensitivity of mononuclear cells (MNCs) to glucose and increase the production of ROS and inflammatory factors by promoting the preactivation of MNCs (48). At the same time, TNF-α, as a mediating factor of IR, activates the inflammatory pathway in insulin target cells to cause serine phosphorylation of IRS-1, resulting in defects in the insulin signaling pathway; interferes with insulin activity; hinders the uptake of glucose by muscle and adipose tissue; and causes IR. The sensitivity of tissue to insulin decreased in the IR state, and compensatory hyperinsulinemia occurred. High-level insulin stimulates the proliferation of theca-interstitial cells, increases the production and secretion of testosterone, reduces the serum SHBG level, improves the bioavailability of serum androgen, and further aggravates HA. IR can also promote liver glycogen synthesis and adipose tissue mobilization so that serum free fatty acid (FFA) levels increase (49). High free fatty acids and a high glucose state promote the production of ROS, and OS imbalance is further aggravated. OS interacts with HA and IR, forming a vicious cycle of mutual promotion and participating in the occurrence and progression of PCOS.

### Overweight/obesity

Overweight/obesity is one of the common clinical manifestations of PCOS, and 40-70% of PCOS patients having obesity (50). Relevant evidence indicates that metabolic abnormalities tend to appear to the overweight/obese PCOS patients (especially abdominal obesity) compared to the PCOS patients with normal BMI (51). Overweight and obese PCOS patients have a considerable amount of macrophage infiltration in their adipose tissues, which has a significantly positive correlation with the severity of obesity (52). Adipose macrophages are highly pro-inflammatory in property; they raise levels of oxidatively active substances and promote the development of OS and low-grade inflammatory states (53). Pro-inflammatory factors generated by macrophages work on adipocytes; therefore, more macrophages accumulate, thus a positive feedback mechanism is formed, followed by an aggravated OS and low-level inflammation. In addition, these pro-inflammatory factors can activate the pathway of inflammation inside insulin target cells thus block the signal transduction of insulin. Furthermore, abnormal lipid metabolism is also a clinical manifestation in overweight/obese population, the FFA level increase causes IR by activating serine kinase among insulin target cells and interfering with insulin signal transduction (39). The adipocytes secreted by overweight/obese PCOS patients, such as adiponectin, resistin, leptin, and other adipokines are strongly correlated to IR (54), leading to HA in an indirect way, and HA can cause abdominal fat accumulation (55). As a consequence, a vicious circle forms causing more serious obesity. Therefore, overweight/obesity caused by inappropriate diet and insufficient exercise can result in PCOS, and the vicious cycle of IR, HA and OS may promote the development of PCOS. Relevant evidence reveals that an appropriate diet, with antioxidants addition and bariatric surgery can be beneficial for those overweight/obese patients with OS imbalance (56–58).

### Effect of oxidative stress, Hyperandrogenemia, and insulin resistance on ovulation disorder in polycystic ovary syndrome

As the basis of follicular growth and development, the follicular fluid microenvironment is particularly important in determining follicular quality. There are various sources of reactive oxygen species in the follicular fluid microenvironment. Related evidence shows that OS markers are expressed in human ovaries in normal cycles (59, 60). In normal follicular fluid, oxidation occurs in a relatively balanced state; that is, there are not only ROS at the physiological level in follicular fluid but also a variety of antioxidant enzymes and non-enzymatic oxidants (61). As the main energy supply organ in follicular fluid, mitochondrial activity is closely related to follicular quality. In the OS-activated state, the follicular fluid of PCOS patients produces excessive ROS, resulting in an imbalance of oxidation/antioxidation in the microenvironment and damaging the function of mitochondria in the follicular fluid (62). Dysfunctional mitochondria arrest and degrade oocytes during meiosis, directly damage oocytes, and cause follicular apoptosis, resulting in ovulation disorders (63). In addition to the changes in the follicular fluid microenvironment, abnormal follicular growth and development are the primary causes of PCOS ovulation disorder. Ovulation disorder in PCOS is characterized by antral follicle growth arrest, and its mechanism is not yet clear. At present, it is believed that the causes can be divided into two aspects: early developmental abnormalities of endogenous follicles independent of gonadotropins and endocrine disorders, such as excessive proliferation of antral follicles caused by HA and IR and follicular development arrest (64). Abnormal early follicular development leads to the inability to choose the dominant follicles, which is a major cause of PCOS ovulation disorder. HA can stimulate the initiation of early follicular development by increasing the expression of IGF-1 receptor, resulting in excessive proliferation of antral follicles and an increase in the proportion of small follicles growing in the ovary (65–67). Hyperinsulinemia and/or high levels of LH synergistically improve the sensitivity of granulosa cells to LH, leading to premature differentiation of granulosa cells and an imbalance in LH/FSH levels, so that FSH deficiency destroys the choice of dominant follicles, leading to the accumulation of small antral follicles (68, 69); at the same time, the premature response of small antral follicles to high LH promotes the early terminal differentiation of follicles (70). In the ovary, the proliferation and differentiation of granulosa cells play an important role in the development of follicles. In granulosa cells before ovulation, FSH regulates glucose conversion into glycogen storage through the IRS-2/PI3K/Akt2 pathway, which plays an important role in the occurrence, development and ovulation of follicles (71). In PCOS, high LH can cause FSH signaling pathway defects and interfere with glucose uptake and glycogen synthesis in granulosa cells, resulting in follicular growth arrest and ovulation disorders (72). In addition, a new study found that HA can increase OS imbalance and low-level inflammation in the ovary and upregulate the expression of inflammatory factors by activating NLRP3 inflammatory bodies, thereby inducing pyroptosis of ovarian granulosa cells, damaging follicular function and leading to ovulation disorders (73).

**FIGURE 1:**
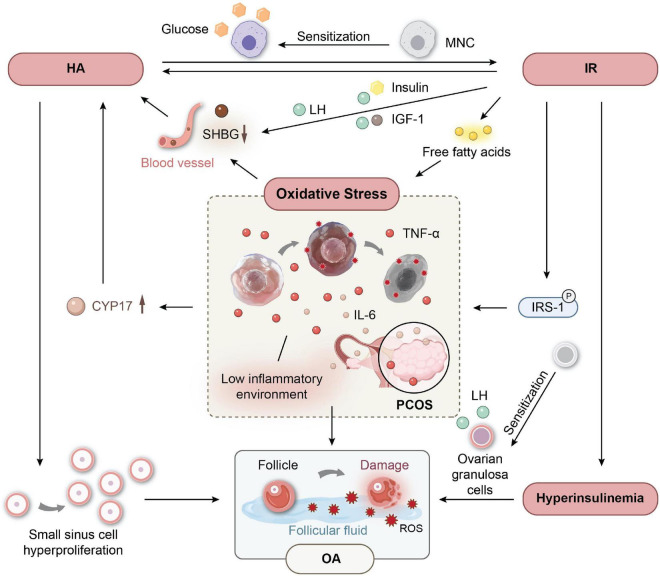
The vicious circle between oxidative stress (OS) and Hyperandrogenemia (HA), insulin resistance (IR) leads to ovulation disorder in (polycystic ovary syndrome) PCOS.

OS, HA and IR play an important role in the development of PCOS, and the vicious cycle between them leads to and aggravates ovulation disorder in PCOS (Figure 1). The above evidence explains the related mechanism of OS, HA and IR in ovulation disorders in the physiological mechanism of PCOS but does not clarify whether OS can cause PCOS ovulation disorders in other ways, such as inducing immune dysfunction or regulating gene expression. Therefore, we need more evidence to explore the mechanism of OS on PCOS ovulation disorder from different perspectives.

### The application of antioxidants in polycystic ovary syndrome

Based on a large number of studies confirming that PCOS patients do exhibit varying degrees of OS, antioxidant therapy may become an important direction for the treatment of PCOS (74) (Table 1). At present, there have been clinical studies on the use of antioxidants in patients with PCOS. Relevant evidence shows that antioxidants can effectively reduce the levels of OS and inflammatory markers in PCOS, improve the antioxidant capacity of the body and improve the OS imbalance. Jamilian et al. (75) found in clinical trials evaluating the effects of vitamin D and omega-3 fatty acids on PCOS patients that the combined use of vitamin D and omega-3 in the treatment of PCOS could significantly reduce the levels of serum total testosterone and serum high-sensitivity C-reactive protein (hs-CRP) and MDA, significantly reduce the expression of the IL-1 gene and significantly increase the level of serum total antioxidant capacity (TAC). In addition, Jamilian et al. (76) evaluated the effect of combined vitamin E and omega-3 supplementation on PCOS patients and found that the combined application of vitamin E and omega-3 could significantly reduce the expression of the IL-8 and TNF-α genes and upregulate the expression of peroxisome proliferator-activated receptor-γ (PPAR-γ). In the above two experiments, the OS status of PCOS patients was significantly improved. In another experiment, Afshar et al. (77) found that the combined application of magnesium and zinc can improve serum TAC and significantly downregulate the expression levels of the proinflammatory cytokines IL-1 and TNF-α so that the OS level of PCOS patients was significantly improved. Rahmani et al. (78) found that in their study, the expression levels of the proinflammatory cytokines IL-1, IL-8, and TNF-α in PCOS patients treated with CoQ10 were significantly downregulated in their experimental group. Mousavi et al. (79) found in a study of the effects of magnesium and/or melatonin on the metabolism of PCOS women that melatonin significantly reduced the level of TNF-α, and the combination of magnesium and melatonin significantly increased the level of TAC. In addition, studies have confirmed that antioxidants can not only reduce OS levels but also improve the clinical symptoms of PCOS patients. Shokrpour et al. (80) found in a study on the treatment of PCOS with magnesium and vitamin D that the combined application of magnesium and vitamin D could effectively reduce the level of hs-CRP and the incidence of hairy diseases, significantly increase the levels of TAC and NO, and reduce the OS level of the body. In another experiment, Gharaei et al. (81) found that astaxanthin treatment significantly increased TAC and increased the expression of nuclear factor E2-related factor 2 (Nrf2) and heme oxygenase-1 (HO-1) in PCOS. At the same time, MII oocytes and high-quality embryos were significantly increased, but there were no significant differences in MDA, SOD and OS markers in follicular fluid. Antioxidants can also significantly improve the IR status of PCOS patients. Tauqir et al. (82) found in a study of acetyl levocarnitine (ALC), metformin, and pioglitazone in the treatment of PCOS that the combination of ALC in the treatment of PCOS could more effectively reduce the fasting insulin level and significantly improve the levels of serum total testosterone, FSH and LH. Similarly, Kazemi et al. (83) found in a study of ellagic acid in the treatment of PCOS that ellagic acid could reduce the concentrations of serum insulin, MDA and serum total testosterone; downregulate the expression level of TNF-α; and significantly improve the OS and IR status of PCOS patients. In another study, Khorshidi et al. (84) found that quercetin in the treatment of PCOS could significantly reduce the gene expression of serum resistin and peripheral blood mononuclear cells and significantly reduce the levels of serum total testosterone and LH. However, compared with the control group, there was no significant difference in each index of IR in the experimental group. Although the therapeutic effect of antioxidants is not the same, the above test shows that antioxidants are beneficial to the treatment of PCOS and can effectively improve the oxidative stress state of PCOS patients.

**TABLE 1:** Clinical study of antioxidant therapy for female polycystic ovary syndrome (PCOS).

Study	Type of study	Study subject	Sample size	Age (years)	Intervention type	Control group	Duration	Outcome	Conclusion
Jamilian et al. (75)	RCT	PCOS	I = 30 C = 30	I = 26.8 ± 4.4 C = 25.1 ± 3.7	50000IU/2 weeks vitaminD + 2,000 mg/day omega-3	placebo	12 weeks	T, hs-CRP, MDA, IL-1↓ TAC, VEGF↑	beneficial
Jamilian et al. (76)	RCT	PCOS	I = 20 C = 20	I = 22.3 ± 4.7 C = 24.4 ± 4.7	400IU/day vitamin E + 1,000 mg/day omega-3	placebo	12 weeks	IL-8, TNF-α↓ PPAR-γ↑	beneficial
Afshar et al. (77)	RCT	PCOS	I = 30 C = 30	data not shown	500 mg/day magnesium oxide + 440 mg/day zinc sulfate	placebo	12 weeks	hs-CRP, PCO, IL-1, TNF-α↓ TAC↑	beneficial
Rahmani et al. (78)	RCT	PCOS	I = 20 C = 20	I = 24.9 ± 3.7 C = 24.7 ± 5.3	100 mg/day CoQ10	placebo	12 weeks	LDLR, IL-1, IL-8, TNF-α↓ PPAR-c↑	beneficial
Mousavi et al. (79)	RCT	PCOS	I(Melation + Mg) = 22 I(Melation) = 21 I(Mg) = 21 C = 20	I (Melation + Mg) = 28.22 ± 6.38 I(Melation) = 25.57 ± 4.99 I(Mg) = 25.57 ± 4.88 C = 26.20 ± 5.72	6 mg/day melatonin + 250 mg/day magnesium, 6 mg/day melatonin_‵_250 mg/day magnesium	placebo	8 weeks	Hirsutism, TNF-α↓ TAC↑	beneficial
Shokrpour and Asemi (80)	RCT	PCOS	I = 30 C = 30	I = 27.2 ± 7.1 C = 26.0 ± 3.7	250 mg/day magnesium + 400 mg/day vitamin E	placebo	12 weeks	Hirsutism, hs-CRP↓ NO_‵_TAC↑	beneficial
Gharaei et al. (81)	RCT	PCOS	I = 20 C = 20	I = 30.60 ± 4.98 C = 30.45 ± 3.98	8 mg/day AST	placebo	40 days	TAC, Nrf2, HO-1, NQ-1↑	beneficial
Tauqir et al. (82)	RCT	PCOS	I = 72 C = 75	I = 26.96 ± 6.31 C = 25.23 ± 6.06	1,000 mg/day metformin + 30mg/day pioglitazone + 3,000 mg/day ALC	1000mg/day metformin + 30mg/day pioglitazone	12 weeks	FBS, FINS, HOMA-IR, LH↓ ADPN↑	beneficial
Kazemi et al. (83)	RCT	PCOS	I = 30 C = 30	I = 25.74 ± 1.19 C = 26.09 ± 1.53	200 mg/day EA	placebo	8 weeks	FBS, Ins, IR, TC, TG, LDL, MDA, CRP, TNF-α, T, PRL, AMH↓	beneficial
Khorshidi et al. (84)	RCT	PCOS	I = 40 C = 40	I = 29.5 ± 4.2 C = 30 ± 5.5	1,000 mg/day quercetin	placebo	12 weeks	Resistin, T, LH↓	beneficial

## Conclusion

In summary, OS, HA, IR, and ovulation disorders are closely related in PCOS patients. These factors interact to cause and aggravate ovulation disorders in PCOS through the amplifying effects of overweight/obesity. Dietary-induced OS promotes the production and secretion of a variety of inflammatory factors. At the same time, OS can promote the proliferation of theca-interstitial cells, upregulate CYP17, reduce serum SHBG levels, increase androgen secretion, improve the biological activity of serum free testosterone, and cause HA. HA can improve the sensitivity of MNCs to glucose, increase ROS and cause insulin signaling pathway defects, causing IR. IR leads to reduced tissue sensitivity to insulin and compensatory hyperinsulinemia and then stimulates the proliferation of theca-interstitial cells to increase the production and secretion of testosterone. At the same time, it reduces the serum SHBG level, improves the bioavailability of serum androgen, further aggravates HA, and forms a vicious cycle. Overweight/obesity exacerbates this vicious cycle by amplifying features such as HA, IR, and OS. OS imbalance affects the follicular fluid microenvironment, reduces the quality of follicles by damaging mitochondria, and even causes follicular apoptosis. HA and IR affect the growth and development of follicles, causing follicular growth stagnation, hindering the selection of dominant follicles and causing ovulation disorders. OS, HA, and IR interact to form a vicious cycle, leading to ovulation disorders in PCOS. However, the mechanism of OS in PCOS patients with ovulation disorders is not yet fully clear, and further studies are needed to confirm this hypothesis. The clinical application of antioxidant therapy may have a beneficial effect on the increase in OS levels in patients with PCOS, but there is still controversy about this treatment at this stage. More clinical trials are needed to confirm that it provides increasingly favorable evidence for the treatment of PCOS in the future.

## Author contributions

WL and CL wrote the manuscript. QY, YZ, and ML sorted out ideas. HS revised the manuscript. All authors read and approved the final manuscript.

## Abbreviations

PCOS: Polycystic ovary syndrome
OS: Oxidative stress
ROS: Reactive oxygen species
RNS: Reactive nitrogen species
SOD: Superoxide dismutase
GSH: Glutathione
MDA: Malondialdehyde
HA: Hyperandrogenemia
GnRH: Gonadotropin-releasing hormone
ACTH: adrenocorticotropic hormone
LH: Luteinizing hormone
SHBG: Sex hormone binding globulin
IGF-I: Insulin-like growth factor-I
IR: Insulin resistance
IRS: Insulin receptor substrate
TNF- α: Tumor necrosis factor- α
IL-1: Interleukins 1
SOCS3: Suppressor of cytokine signaling 3
CYP17: Cytochrome P450-17 α -hydroxylase
HNF-4 α: Hepatocyte nuclear factor-4 α
MNCs: Mononuclear cells
FFA: Free fatty acid
hs-CRP: High-sensitivity C-reactive protein
TAC: Total antioxidant capacity
PPAR- γ: Peroxisome proliferator-activated receptor- γ
Nrf2: Nuclear factor E2-related factor 2
HO-1: Heme oxygenase-1
ALC: Acetyl levocarnitine
T: Testosterone
VEGF: Vascular endothelial growth factor
PCO: Polycystic ovary
LDLR: Low density lipoprotein receptor
FBS: Fasting blood sugar
FINS: Fasting insulin
HOMA-IR: Homeostatic model assessment for insulin resistance
ADPN: Adiponectin
Ins: insulin
TC: Total cholesterol
TG: Triglyceride
PRL: Prolactin
AMH: Anti-Müllerian hormone.
